# Prescriptions

**Published:** 1895-11

**Authors:** 


CHLOASMA—"LIVER SPOTS."

℞. Hydrargyri ammoniati..gr. iss.

Zinci oxidi puri..........gr. iij.

Olei theobromæ.........ʒxij.

Olei ricini................ʒxij.

Ess. rosæ ................q. s.

M. Sig.:—This ointment is applied morning and evening, after washing the surface wlth soap and water.

ATONIC CONSTIPATION.

℞. Aloes purificat..........gr. v.
Ext. bellad. fol..........gr. iij.
Ext. nucis vom..........gr. v.
Oleoresin capsici........gr. ij.
Ext. physostigmat......gr. v.

Ft. Pil No. XX.

Sig.:—One after each meal, until more than one action occurs daily· Continue reduction by taking half pills, until habit of regular evacuations has been formed.

SYNOVITIS.

℞. Aquæ camphor.
Tinct. opii. deoderat.
Aquæ hamamel dest..... aa ℥ij.

Sig.:—Saturate cloths, and apply to painful part. Keep moist, and cover with oiled silk.

GONORRHŒA.

℞. Hydrarg. chlor. cor....gr. ss-iss
Zinci sulpho-carbolat.gr. ii–x.
Acid. boric............ʒj.
Hydrogen peroxide...fl ℥j.
Aq. destillat..q. s. ad. fl ℥viij.

M. Sig.:—Use as an injection from four to six times a day, immediately after urinating.—*White.*

PILES.

℞. Liq. plumb. subacetati....℥i.
Tr. opii..................ʒiv.

M. Sig.:—Teaspoonful to a wineglass of water, and apply night and morning.—*Allingham.*

FROSTBITE.

℞. Acid. carbolici ........... ʒj.

Tr. iodinii .... ........... fl ʒij.

Acid. tannici ............. ʒj.

Cerat. simplicis .......... ℥iv.

M. Sig.:—Apply two or three times a day.—*Morrow.*

DIARRHŒA WITH CRAMPS.

℞. Tinct. opii. camph.
Tinct. lavendulæ comp.
Tinct. cardomomi comp., aa ℥j.
Aqua menth. pip ........... ℥iij.
Sig.:—Tablespoonful every two or three hours.

GOOD LOCAL ANÆSTHETIC.

℞. Menthol ..................... ʒi.

Chloroform ................. ʒx.

Etheris ...... ........... ʒxv.

Sig.:—Use as spray over field of operation. Anæsthesia lasts from two to six minutes.—*Louisville Medical Monthly.*

